# A Critical Review of Animal Models Used in Acute Myeloid Leukemia Pathophysiology

**DOI:** 10.3390/genes10080614

**Published:** 2019-08-13

**Authors:** Hala Skayneh, Batoul Jishi, Rita Hleihel, Maguy Hamieh, Nadine Darwiche, Ali Bazarbachi, Marwan El Sabban, Hiba El Hajj

**Affiliations:** 1 Department of Experimental Pathology, Microbiology and Immunology, Faculty of Medicine, American University of Beirut, Beirut 1107 2020, Lebanon; 2 Department of Anatomy, Cell Biology and Physiological Sciences, Faculty of Medicine, American University of Beirut, Beirut 1107 2020, Lebanon; 3 Department of Internal Medicine, Faculty of Medicine, American University of Beirut, Beirut 1107 2020, Lebanon; 4 Department of Biochemistry and Molecular Genetics, Faculty of Medicine, American University of Beirut, Beirut 1107 2020, Lebanon

**Keywords:** Zebrafish, Drosophila, rats, mice, NPM-1, FLT3 ITD, ETO-1, IDH1/2

## Abstract

Acute myeloid leukemia (AML) is one of the most frequent, complex, and heterogeneous hematological malignancies. AML prognosis largely depends on acquired cytogenetic, epigenetic, and molecular abnormalities. Despite the improvement in understanding the biology of AML, survival rates remain quite low. Animal models offer a valuable tool to recapitulate different AML subtypes, and to assess the potential role of novel and known mutations in disease progression. This review provides a comprehensive and critical overview of select available AML animal models. These include the non-mammalian *Zebrafish* and *Drosophila* models as well as the mammalian rodent systems, comprising rats and mice. The suitability of each animal model, its contribution to the advancement of knowledge in AML pathophysiology and treatment, as well as its advantages and limitations are discussed. Despite some limitations, animal models represent a powerful approach to assess toxicity, and permit the design of new therapeutic strategies.

## 1. Introduction

Acute myeloid leukemia (AML) is an aggressive and heterogeneous hematological group of neoplasms characterized by increased proliferation of myeloid progenitor cells and a reduced capacity to differentiate. This results in the accumulation of myeloblasts in the bone marrow (BM), which negatively impacts hematopoiesis and leads to BM failure [1]. AML is one of the most common acute leukemia in adults [2]. Its incidence rate is 2.5 per 100,000 cases/year and the median overall survival (OS) is approximately nine months [3]. AML treatment and prognosis largely depend on the patients’ age [4,5,6]. AML was historically divided into eight major groups according to cell morphology and immune phenotype (M0 to M7) [7]. This classification has been revised several iterations since then [8,9,10,11,12]. Exome sequencing in AML patients led to the current classification through identification of more than 20 driver recurrent mutations [13]. These mainly include *Nucleophosmin-1* (*NPM1*), *DNA methyltransferase 3A* (*DNMT3A*), *Fms-like tyrosine kinase-3* (*FLT3*), *isocitrate dehydrogenase* (*IDH*), *Ten–Eleven Translocation 2* (*TET-2*), *Runt-related transcription factor* (*RUNX-1*), *CCAAT enhancer binding protein α* (*CEBPA*), *additional sex comb-like 1* (*ASXL1*), *mixed lineage leukemia* (*MLL*), tumor protein *p53* (*TP53*), *c-KIT* [14]. These mutations dictate the response to treatment, rates of complete remission, disease-free survival, overall survival, and classify AML into three prognostic risk factors (favorable, intermediate, and adverse) (Table 1). 

Animal models provide an excellent tool to understand the biology of pathological mechanisms involved in human diseases. Diverse animal species were used to answer pivotal questions related to disease progression, genetic mutations, immunity, and response to treatment. Among these models, Zebrafish was exploited to generate different mutations mimicking several subtypes of human AML. 

## 2. Zebrafish: Characteristics and Relevance to Human Blood Malignancies

*Danio rerio*, commonly known as Zebrafish, shares genetic and molecular mechanisms of hematopoiesis with humans [15]. This model offers many advantages, including low-cost, optically transparent embryos, high fecundity, rapid embryogenesis, and short gestation time. The genome editing in zebrafish was known since 1970s, when the first transgenic zebrafish was generated by inserting naked linear DNA [16]. Since then, the genetic manipulation of this model evolved to include clustered regularly interspaced short palindromic repeats (CRISPR) technology [17], which renders zebrafish an attractive model for studying specific gene involvement and for drug screening in blood malignancies [18,19,20].

During normal zebrafish hematopoiesis, both the primitive and definitive waves arise from the mesoderm germ layer under the control of the Transforming Growth Factor beta (TGF-β) superfamily proteins, known as bone morphogenic proteins (BMP such as bmp2b and bmp7) [21,22,23]. The generated transient primitive erythroid and myeloid cells are essential for the embryonic development, while the hematopoietic stem cells (HSCs) and progenitor cells (HSPCs) produce blood lineages in the adult fish [24]. In the below section, we will provide an overview of AML models of Zebrafish (summarized in Table 2).

### 2.1. AML Models of Zebrafish

#### 2.1.1. Spi-1: MYST3/NCOA2-EGFP

MYST3 (MOZ) is a member of the MOZ, YBF2, SAS2, TIP60 (MYST) family of histone acetyl-transferases (HAT), while NCOA2 (TIF2) is a member of the p160 HAT family [25,26,27,28]. The first AML model in Zebrafish was created by expressing the fusion protein, MYST3/NCOA2 (MOZ/TIF2). This fusion targets hematopoietic cells under the control of *spi-1 (pu.1)*, an early myeloid promoter [29]. pu.1 is an ETS-domain transcription factor expressed in both immature lymphoid/hematopoietic cells and myeloid cells during zebrafish hematopoiesis [30]. Cells expressing pu.1 differentiate into myeloid progeny, whereas cells with low pu.1 expression shift to the erythroid fate [31]. After an extended latent period, a small percentage of transgenic fish developed AML [29]. These animals presented with an extensive invasion of kidneys by myeloid blast cells, proving the oncogenic potency of *MYST3/NCOA2* fusion gene [29]. Although this model is useful as a chemical library screen, especially for compounds that target epigenetic regulation of gene expression [29], the long latency and low incidence waned the enthusiasm for its use.

#### 2.1.2. hsp70: AML1-ETO

A chromosomal translocation between chromosomes 8 and 21 (t(8;21)(q22;q22)) occurs in 12–15% of AML patients [32]. This chromosomal rearrangement yields a fusion transcription factor encoding AML1 (RUNX1) linked to ETO, forming the AML1-ETO fusion product [33,34,35]. This translocation was introduced under the control of the heat shock promoter *hsp70* in zebrafish embryos (*hsp70: AML1-ETO*). Transgenic Zebrafish recapitulated the human AML features, at both the cytological and transcriptional levels [36]. The expression of this fusion protein led to the accumulation of non-circulating hematopoietic cells, whereby the intermediate cell mass was enriched with myeloperoxidase positive neutrophils and morphologically immature hematopoietic blasts [36]. The disruption of definitive hematopoiesis led to switching the cells fate from the erythroid to the myeloid lineage [36]. Overexpression of the transcription factor reversed the observed phenotypes, implicating scl, as major player downstream of AML1-ETO [36]. This model enabled the screening of a small molecule library and discovery of compounds that antagonize the activity of AML1-ETO in the hematopoietic progenitor cells (HPCs) [36]. Inhibition of COX-2 and β-catenin signaling antagonized AML1-ETOs effects on HPCs differentiation and may have implications in human AML [37].

#### 2.1.3. MYCN: HSE: EGFP

MYCN (N-myc) proto-oncogene is upregulated in many types of hematological malignancies [38,39] including 20 to 40% of pediatric AML patients [40]. To unravel the molecular and transcriptional networks by which MYCN induces malignancy, Shen et al. established a transgenic embryonic zebrafish model, Tg (*MYCN: HSE: EGFP*), expressing the murine MYCN under a heat shock promoter [41]. MYCN overexpression induced immature myeloid blast cell expansion and reprogrammed the hematopoietic cell fate through MYCN downstream-regulated gene 1b (ndrg1b) and other lineage-specific hematopoietic transcription factors regulation [41]. The primitive hematopoiesis was enhanced through scl and lmo2 upregulation. Furthermore, erythroid differentiation was blocked through downregulation of gata1, while myelopoiesis was promoted by pu.1 overexpression [41]. This model presents a high AML incidence (∼75% of transgenic zebrafish) and a rapid onset occurrence, providing a platform for whole-organism chemical suppressor screens, to identify compounds that can reverse MYCN function in vivo [41].

#### 2.1.4. FLT3-ITD and NPM1c+ Models in Zebrafish

FLT3-ITD and NPM1 are two major players in defining the prognosis and response to treatment in AML patients. FLT3 is a tyrosine kinase receptor that plays a major role in hematopoiesis through the regulation of proliferation, differentiation, and apoptosis of HPCs [42]. It is highly expressed on leukemic blasts of 70–100% of AML patients [43,44]. Several mutations occur in the FLT3 receptor, the most common of which leads to an internal tandem duplication (ITD) [45]. FLT3-ITD occurs in 20% of AML patients and is strongly associated with poor prognosis [46,47]. NPM1, a shuttling protein between the nucleoplasm and the cytoplasm, plays several roles, notably ribosomal biogenesis [48,49]. NPM1 is mutated (NPM1c+) in around 30% of AML patients with normal karyotype [50]. NPM1c+ is continuously translocated to the cytoplasm contributing to leukemogenesis [50].

FLT3-ITD plays a role in embryonic primitive and definitive hematopoiesis in zebrafish. Transgenic zebrafish embryos with human FLT3-ITD showed expansion and clustering of myeloid cells [51]. Thus far, the impact of FLT3-ITD on adult zebrafish remains underexplored.

Bolli et al. generated a transgenic zebrafish model expressing NPM1c+, which perturbed primitive hematopoiesis by promoting the early expansion of pu.1+ myeloid cells [52]. This phenotype was even more pronounced in a p53-deficient background [52]. An increase in the number of gata1+/lmo2 indicating expansion of erythro-myeloid progenitors (EMPs) was also observed. These EMPs highly expressed both c-myb and CD41 but not RUNX1, suggesting a disruption of definitive hematopoiesis where these cells could be the main target of NPM1c+. This model provides a tractable in vivo system for the study of the mechanisms through which hematopoietic development is perturbed in the presence of NPM1c+ [52].

Transgenic zebrafish models expressing either human FLT3-ITD or NPM1 proteins under the control of *pu.1* promoter were also generated [53]. For that purpose, *spi-1*: FLT3-ITD-2A-EGFP/CG2 expressing mutant FTL3-ITD and *spi-1*: NPM1-Mut-PA/CG2 expressing mutant NPM1 constructs were designed. This double mutant transgenic fish (FLT3-ITD/NPM1.Mut) exhibited an accelerated rate of myeloid leukemogenesis [53]. By the age of six months, around 66% of the transgenic fish produced significantly increased precursor cells in the kidney marrow along with dedifferentiated myeloid blasts [53].

#### 2.1.5. Spi-1: CREB-EGFP

The cAMP response element binding protein (CREB) plays a major role in hematopoiesis through the regulation of proliferation and differentiation of myeloid progenitor cells [54]. Overexpression of CREB is associated with immortalization, growth factor-independent proliferation and blast-like phenotype in BM progenitor cells [55]. CREB is highly expressed in BM samples of both adult and pediatric AML patients [56]. Tregnago et al. generated a transgenic zebrafish model (*spi-1: CREB-EGFP*) expressing the *CREB* gene downstream *pu.1* promoter in the myeloid cell lineage. CREB overexpression resulted in upregulation of erythroid and myeloid genes, altering primitive hematopoiesis. Among adult transgenic zebrafish, 80% of the fish developed AML after 9–14 months through the blockage of myeloid differentiation [57]. These fish showed aberrant expression of a set of 20 genes in common with pediatric AML. The most intriguing is the CCAAT-enhancer-binding-protein-δ (C/EBPδ) that acts downstream CREB. It resulted in impaired myeloid differentiation that could be reversed through inhibition of the CREB-C/EBPδ axis. These findings are complementary with the data obtained by screening for CREB and C/EBPδ in pediatric AML patients, offering an opportunity to test for novel therapeutics through this model [57].

#### 2.1.6. Spi-1: SOX4-EGFP

SOX4 is a transcription factor belonging to the SOX (Sry-related high-mobility groupbox) family [58]. In AML patients, SOX4 overexpression results in poor prognosis and short overall survival [59]. SOX4 was reported to contribute to the leukemic phenotype of C/EBPα mutant AML in murine models as well as in human AML. C/EBPα protein typically inhibits the self-renewal of leukemic cells and restores cellular differentiation. SOX4 overexpression results in C/EBPα inactivation, enabling leukemic cells proliferation and AML development [60,61].

Lu et al. generated a transgenic zebrafish model Tg (spi-1:SOX4-EGFP) expressing SOX4 protein downstream the spi-1 myeloid promoter. Early developmental stages of transgenic zebrafish did not reveal a difference of expression of SOX4. However, by the age of five months, Tg (spi-1:SOX4-EGFP) zebrafish kidneys started showing mild vacuoles in the renal tubule which evolved into effacement, distorted structure, and increased infiltration of myeloid cells by the ages of 9 and 12 months. A higher number of myeloid progenitor cells and excess blast cells with focal aggregation were observed in the kidney marrow blood cells of 9-, 12-, and 15-months old fish but not younger ones, highlighting that myeloid transformation is age-dependent [59].

#### 2.1.7. IDH 1/2 Mutation

Mutations identified in a family of enzymes involved in the citric acid cycle, isocitrate dehydrogenases 1/2 (IDH1/2), account for 16% of AML patients [62]. These mutations substitute arginine residue almost exclusively at codon 132 in IDH1 (IDH1-R132H) and codons 140 and 172 in IDH2 [62]. To study the involvement of IDH in AML, *zidh1* was either suppressed or deleted and resulted in the blockage of differentiation and accumulation of early myeloid progenitor cells, while decreasing macrophage and natural killer progenitor cells [63]. The importance of IDH1 mutation was asserted when plasmids of IDH1-R132H were injected into zebrafish embryos [63]. An increase in 2-hydroxyglutarate (2-HG) level, a reduction of 5-Hydroxymethylcytotsine (5-hmC), and an expansion of myelopoiesis were obtained in these embryos. A human IDH1-R132H–specific inhibitor significantly ameliorated both hematopoietic and 2-HG responses in human but not zebrafish IDH1 mutant expression [63]. This result is not surprising and highlights some of the drawbacks using Zebrafish as a model for human diseases. On the other hand, studies on *zidh2* were restricted to the regulation of embryonic hematopoiesis in zebrafish but with no relevance to the human AML [63].

Even with the drawbacks of not possessing many mammalian-like organs, zebrafish still provides an excellent, affordable, and rapid platform for evaluating several aspects of AML. The variations in the biological microenvironment might impede drug delivery and performance in humans. Additionally, zebrafish are ectothermic (cold-blooded), so their physiology is not identical to humans, which might affect enzyme kinetics and metabolism. The genetic diversity detected between individual zebrafish belonging to the same strain confounds data and could be misleading [64]. The sparsity of reagents to study zebrafish at the molecular level is contrasted by the abundance of mouse-specific reagents.

## 3. Rodent Models

Due to the complexity and heterogeneity of AML in humans, rodent models have been instrumental in providing a platform for answering pivotal questions related to AML pathogenesis, disease progression, and developing new effective therapeutic approaches. Among these models, rats and mice represent the closest accepted mammalian models to AML.

### 3.1. Rats

Several transplantable leukemia rat models were established using carcinogens, radiations, and pollutants [65,66,67].

#### Transplantable Rat Models

Acute Myeloid Leukemia/ Chronic Meylogenous Leukemia (AML/CML) leukemia: Repeated intravenous injections of 7, 12-dimethylbenz (a) anthracene (DMBA) into WOP/H-Onc strain or Wistar/H-Onc strain, induced leukemia in 10% of the rats in 5–9 months. This leukemia has myeloid characteristics as revealed by hematological and histological examination, as well as infiltration of myeloid blasts into several organs (BM, liver, spleen, and lymph nodes). This myeloid nature showed similarities with both human CML (as demonstrated by high peroxidase and Sudan black B positive cells and reduction in alkaline phosphatase positivity) and human AML (non-specific esterase activity, highly reduced in the peripheral blood but slightly reduced in BM). These findings do not support the use of these rats as an exclusive AML model [68].

Brown Norwegian Myelogenous Leukemia (BNML): The transplantable promyelocytic leukemia in BN rat (BNML) was first described in 1971. This slow growing leukemia shares many common characteristics with AML, including the disappearance of normal hematopoiesis [69]. Similarities in in vitro colony forming assays between AML patients and BNML rats validated it as a model for AML [70,71]. Several therapeutic modalities were optimized using this model; these include the combination of anthracyclines, [72,73] Ara-C, [74,75], 4′-(9-acridinylamino) methanesulfon-m-anisidide (AMSA) [76], and other therapeutics [77,78,79]. One of the most significant advantages in the BNML model is its contribution to the improvement of minimal residual disease (MRD) detection by karyotyping [80] and multidimensional flow cytometry [81,82].

### 3.2. Mice

Mice offer an invaluable model due to their small size, cost-effectiveness, and easy maintenance, availability of research tools, and ease of manipulation to produce and recapitulate several human diseases, including cancer. Since hematopoiesis in mice has been well characterized, they provide a reasonably reproducible model to study AML pathogenesis and potential therapies. Murine AML models include induced, transgenic animals, and humanized mouse models (Table 3) among others.

#### 3.2.1. Chemically-Induced Model

AML models were generated using the L1210 and p388 cell lines, isolated from DBA/2 mice chemically exposed to the carcinogen 3-methylcholantrene [83]. These models were transplantable and provided a platform for testing chemotherapeutic drugs, studying their kinetics, and evaluating their anti-leukemic effectiveness [84]. The L1210 model was used to screen anthracyclines [85] and antimetabolites [86,87] including Cytarabine [88]. The p388 model was used to investigate the efficacy of natural products as topoisomerase II inhibitors [89]. These models allowed significant improvement in the treatment of AML, including the currently used Cytarabine [90]. The main limitation of using these animal models is the induction of more lymphoid than myeloid leukemia, and the needed prolonged exposure to those carcinogens to develop leukemia [91].

#### 3.2.2. Radiation-Induced Model

The correlation between radiation and leukemia was established in patients exposed to x-rays, and survivors of nuclear attacks. Among this cohort of subjects, children presented mostly with ALL, whereas adults were more prone to CML and AML [92,93,94,95]. All established radiation-induced AML models carry deletions on chromosome 2, where the hematopoietic transcription factor *Sfpi1/pu.1* is located [96].

##### RF Model

The RF strain was developed by Furth in 1933 at the Rockefeller Institute [97]. In this model, myeloid leukemia was developed following exposure to fission neutron irradiation or gamma irradiation [98]. In the RF model, a single dose of ionizing radiation-induced myeloid leukemogenesis in 4–6 months, with symptoms reminiscent to human AML [99]. Flt3-ITD mutations were identified in 10% of RF mice [100], which correlates with the occurrence of this mutation in human AML [101].

##### SJL/J Model

This model is characterized by high spontaneous frequency of reticulum cell neoplasm type B at an early age [102]. The radiation-induced AML in this model is similar to the secondary human AML occurring after irradiation of Hodgkin disease patients [103]. The efficient development of AML required the addition of promoting factors, such as corticosteroids and growth factors, colony stimulating factor CSF-1, known to be high in AML patients [104].

##### C3H/He and CBA Models (CBA/Ca, CBA/Cne, and CBA/H)

These models were generated in 1920, by cross-breeding Bragg albino with DBA mice. While C3H/He was specifically selected for the high incidence of mammary tumors [105], CBA was selected for a lower incidence of mammary tumors. The C3H/He was detected 24 h after irradiation in BM cells; this indicates that chromosomal 2 alteration is responsible for the initiation of myeloid leukemogenesis [106]. CBA showed chromosome 2 and 4 aberrations [107,108]. Moreover, an 8% decrease in DNA methylation was observed after exposure to radiation. This hypomethylation played a role in leukemogenesis [109]. The CBA model is considered the most favorable model in radiation-inducedAML because of low spontaneous leukemia incidence (0.1 to 1%), high incidence of AML after exposure to radiation or benzene, with lower latency, compared to other models, and more importantly, it mimics human AML at the cytological, histopathological, and molecular levels.

#### 3.2.3. Virally Induced Leukemia Models

Murine leukemia viruses (MuLV) induce non-B and non-T cell leukemia in mice [110,111] and are considered among the simplest retroviruses that shed light on the pathogenesis of leukemia [112,113]. A model was created by injecting cell-free filtrates, including replication-deficient spleen focus forming virus (SFFV) and a replication-competent Friend MuLV [114,115]. It was noticed that the same infection of MuLV induces several subtypes of AML (Table 4), resembling French–American–British (FAB) classification of human AML [116]. Furthermore, MuLV-induced AML led to the discovery of several genes with a significant role in the regulation of growth, death, lineage determination, and development of hematopoietic precursor cells [117]. MuLV induced AML is considered a critical landmark for understanding the pathogenesis of human AML, since it unraveled relevant unknown oncogenes to leukemogenesis (Table 4).

#### 3.2.4. Transposon Models

Sleeping Beauty (SB) transposon is an insertional mutagenesis system, allowing overexpression or inactivation of specific genes depending on the transposon orientation and integration site [118,119]. SB consists of a mobilized piece of DNA, transposon, and a transposase enzyme [120]. In a transgenic animal with a humanized NPM1c+ knock-in allele, this system enhanced the incidence and onset of AML in NPM1c+ mice [121]. An advantage of this model was the identification of mutations in leukemia genes [121].

#### 3.2.5. Transgenic Models: Single Mutation

##### PML-RARα t(15;17)

Acute promyelocytic leukemia (APL) is a subtype of AML, characterized by t(15;17) chromosomal translocation, resulting in the promyelocytic leukemia-retinoic acid receptor α (PML-RARα) fusion protein [122,123]. PML-RARα was expressed in three mouse models under the myeloid regulatory promoters. Under the *CD11b* promoter, transgenic mice showed abnormal myelopoiesis and increased radiation sensitivity, however, did not develop any leukemia [124]. Mice expressing the transgene under the human cathepsin G (*HCG*) and human MRP8 (*hMRP8*) promoters [124,125,126] developed APL phenotypes after a long period of latency [125,126]. These two models recapitulated the remissions seen after all trans-retinoic acid (ATRA) treatment in human APL [125,126].

##### AML1-Eight-Twenty One Oncoprotein

AML1-Eight-Twenty One oncoprotein (ETO) chimeric product, encoded by the t(8;21), occurs in around 12–15% of AML [32]. Knock-in mice expressing AML1-ETO is embryonic lethal due to the complete absence of liver-derived definitive hematopoiesis [127,128]. Embryonic livers contained dysplastic multilineage hematopoietic progenitors that had an abnormally high self-renewal capacity in vitro, a phenotype typical of leukemic cells [129]. To bypass the embryonic lethality, inducible transgenic models were generated. These mice expressed AML1-ETO in their BM progenitor cells [130,131]. Although abnormal maturation and proliferation of progenitor cells were observed, mice failed to develop leukemia [130,131]. Expression of AML1-ETO under the control of *hMRP8* promoter was unable to develop AML until their exposure to a robust DNA-alkylating mutagen, *N*-ethyl-*N*-nitrosourea [132]. To further enhance AML development, this mouse model was modified by either the expression of other factors or mutations in tyrosine kinases such as c-KIT, FLT3-ITD, or the TEL- platelet-derived growth factor receptor β (PDGFbR) [133,134].

##### CBFB-MYH11

The beta subunit of the core binding complex (CBFB) is a heterodimeric core-binding transcription factor, with a critical role in hematopoiesis [135]. CBF products, due to chromosomal translocations, account for approximately 25% of pediatric and 15% of adult AML patients [136]. The translocation Inv(16) (p13;q22) is a result of the binding of CBFB subunit to the tail region of the smooth muscle myosin heavy chain (*SMMHC*) gene, MYH11 [137]. The resulting fusion protein (CBFB-MYH11) competes with the binding of CBF to target genes, disrupting transcriptional regulation, thus contributing to leukemic transformation [137]. Similar to embryos with homozygous mutations in AML1 [128], knock-in embryonic mice (*Cbfb^+/Cbfb-MYH11^*) lacked definitive hematopoiesis and died during gestation [138]. Chemically or retrovirally induced mutations in heterozygous *CBFB-MYH11* adults led to AML development [138,139]. A conditional knock-in mouse model expressing *CBFB-MYH11* fusion protein in adult mice (*Cbfb^+/56M^)* was also generated [140] and led to AML development in 90% of the mice within five months [140].

##### Mutant Nucleophosmin-1 (NPM1c+)

Mutations in the *Nucleophosmin-1 (NPM1)* gene represent one of the most frequent genetic aberrations in AML [141] and account for 30% of AML patients [50]. Transgenic mice harboring the *NPM1c+* mutation developed myeloproliferation in BM and spleen, supporting a role of NPM1c+ in AML [142]. Chou et al. generated a knock-in transgenic mouse model by inserting the most frequent mutation, TCTG called mutation A, in the C-terminus of wt-NPM1 [143]. Mice homozygous for the transgene encountered embryonic lethality, whereas one-third of the heterozygotes (*Npm1wt/c+*) developed the fetal myeloproliferative disease but not AML [143]. Conditional expression of *NPM1c+* with further genetic manipulations resulted in two models [121,144]. In one model, one-third of the transgenic mice developed leukemia after a long period of latency associated with AML features [144]. In the other model, the expression of humanized NPM1c+ in the hematopoietic stem cells caused *HOX* overexpression, enhanced self-renewal, and expanded myelopoiesis [121].

##### Fms-Related Tyrosine Kinase 3 Internal Tandem Repeats

The second most common genetic aberrations in de novo AML patients occur in the fms-related tyrosine kinase 3 internal tandem repeats (*FLT3-ITD*) gene on chromosome 13. These associate with poor prognosis and short overall survival (OS) [145]. A transgenic mouse model expressing FLT3-ITD under the *vav* hematopoietic promoter was created [146]. The majority of transgenic mice developed a myeloproliferative syndrome (MPS) characterized by megakaryocytic hyperplasia and thrombocytosis but not AML [146]. In FLT3-ITD knock-in mice, loss of FLT3 wild-type allele contributed to myeloid expansion and aggressiveness of the MPS disease [147]. Several other models expressing this mutation also revealed MPS but not AML [148,149].

##### Mixed Lineage Leukemia (MLL)

The translocation t(9;11)(p22;q23) produces the fusion product MLL-AF9 [150,151]. In one model, embryonic stem cells were generated from an in-frame fusion of AF9 with exon 8 of mouse MLL [152]. Other models conditionally expressed MLL-AF9 [153]. These models developed only AML despite the widespread activity of the MLL promoter [152,153]. Conditional expression of MLL-AF9 in long-term hematopoietic stem cells (LT-HSC) produced aggressive AML with extensive tissue infiltration, chemo-resistance, and expressed genes related to epithelial-mesenchymal transition in solid cancers [154]. MLL early introduction results in abnormalities of myeloid cell proliferation and differentiation [155]. Moreover, HOXa9 was found to be essential for the MLL-dependent leukemogenesis in vivo [156].

The translocation t(4;11)(q21;q23) produces the fusion product MLL-AF4. This translocation is associated with pro-B-ALL and rarely AML [157]. Although several models have been established for this translocation, only few models resulted in AML. MLL*-AF4* models generated using both a knock-in [158] and *Cre*-inducible invertor model [159] produced large B-cell lymphoma rather than the immature acute leukemia observed in humans [158,159]. The MLL-AF4 expression in hematopoietic precursors, during mouse embryonic development, developed long latency B-cell lymphoma [159,160]. Furthermore, MLL-AF4 knock-in followed by in vitro inducible transduction generated mice with both AML and pre-B-ALL as well as a few MLLs [161].

Leukemia with the t(11;19)(q23;p13.3) translocation express MLL-ENL fusion proteins capable of malignant transformation of myeloid and/or lymphoid progenitor(s). Immortalized cells containing MLL-ENL proviral DNA or enriched primary hematopoietic stem cells transduced with MLL-ENL induced myeloid leukemia in syngeneic and SCID recipients [162]. Using an in vitro B-cell differentiation system, retroviral transduction of *MLL-ENL* generated a leukemia reminiscent of human MLL-ENL ALL [163]. Other models expressed MLL-ENL-ERTm, the ligand-binding domain of the estrogen receptor modified to specifically recognize synthetic but not endogenous estrogens, using retroviral transduction approach [164]. Several other models were generated encountering more mutation along with MLL-ENL [165,166].

##### IDH 1/2

A conditional knock-in mouse model was created by inserting the mutated human IDH1 (R132H) into the endogenous murine *idh1* locus. IDH1 (R132H) was expressed in all hematopoietic cells under the *vav* promoter (vav-KI mice) or specifically in cells of the myeloid lineage (LysM-KI mice) [167]. Transgenic mice showed increased number of early hematopoietic progenitors and developed splenomegaly and anemia with extramedullary hematopoiesis, characteristics of a dysfunctional BM niche, along with partial blockage in myeloid differentiation [167]. Moreover, LysM-KI cells have hypermethylated histones and changes to DNA methylation similar to those observed in human *IDH1*- or *IDH2*-mutant AML, demonstrating the induction of leukemic DNA methylation signature in the mouse model [167].

#### 3.2.6. Transgenic Models: Compound Transgenic Mouse Models

##### K-RAS-G12D + PML-RARα

4% and 10% of APL patients with PML-RARα fusion had oncogenic *N-RAS* and *K-RAS* mutations, respectively [168,169]. The conditional expression of oncogenic K-RAS and PML-RARα in mice induced a rapid-onset and highly penetrant, lethal APL-like disease [170].

These mice may be used to test for the therapeutic efficacy of inhibitors of RAS post-translational modifications and RAS downstream signaling [170].

##### N-RASD12 + BCL-2

N-RAS, a protein belonging to the family of RAS GTP-ases, is mutated in patients at risk of leukemic transformation after chemotherapy and/or radiotherapy [171]. *N-RAS* mutation at codon 12 is the most frequent abnormality in myelodysplastic syndromes (MDS), associated with AML transformation and poor OS [172]. B-cell lymphoma 2 (BCL-2) protein is an apoptosis regulatory protein. BCL-2 is overexpressed in AML patients [173], which blocks the differentiation of myeloid progenitors [174]. Both mutants have been previously identified as risk factors for AML in MDS patients [172].

Two murine models of initiation and progression of human MDS/AML were generated [175]. The transplantable model expressing hBCL-2 in a primitive compartment by mouse mammary tumor virus–long terminal repeat *(MMTVtTA /TBCL-2/NRASD12)* represents human MDS, whereas the constitutive *MRP8 [BCL-2/NRASD12]* model is closer to AML [175]. Both models showed expanded leukemic stem cell (Lin^−^/Sca-1^+^/c-Kit^+^) populations. hBCL-2 is observed in the increased RAS-GTP complex within the expanded Sca-1^+^ compartment [175]. The difference of hBCL-2 oncogenic compartmentalization associates with the pro-apoptotic mechanisms in MDS and the anti-apoptotic in AML mice [175]. Downregulation of hBCL-2 in MDS mice partially reversed the phenotype and prolonged survival; however BM blasts and tissue infiltration persisted [175]. This model revealed that the two candidate oncogenes *BCL-2* and mutant *N-RAS* can cooperate to give rise to malignant disease with a penetrance of around 80% and a latency period of 3 to 6 months [175].

##### Mixed Lineage Leukemia-Partial Tandem Duplication + FLT3-ITD

Mixed lineage leukemia-partial tandem duplication (*MLL*-PTD) is expressed in 5 to 7% of cytogenetically normal (CN)-AML patients [176,177]. Approximately 25% of these patients have constitutive activation of FLT3-ITD, conferring a poor prognosis [178]. To recapitulate the *Mll^PTD/WT^:flt3^ITD/WT^* AML found in humans, a double knock-in mouse model was generated by expressing these two mutated genes under their respective endogenous promoters [179]. After a period of latency, this model developed AML with a short life span, extensive extramedullary involvement, and increased aggressiveness [179]. Reminiscent of this subtype of AML in humans, these transgenic mice have normal chromosomal structures, reduced *MLL-WT* expression, loss of *FLT3*-WT, and increased total *FLT3* expression [179,180,181,182]. Moreover, increased *HOXA9* transcript levels were observed, rendering this model valuable for the assessment of epigenetic modifying agents combined with tyrosine kinase inhibitors [179].

##### NUP98-HOXD13 + FLT3-ITD

The chromosomal translocation t(2;11)(q31;p15) leads to the fusion of Nucleoporin *(NUP98)*, a structural component of the nuclear pore complex, to the homeobox protein NHD13 (HOXD13), inducing leukemogenesis [183]. *NUP98-HOX* fusions are observed in human and murine MDS [184]. Clinical and experimental evidence demonstrated that high rate of FLT3-ITD mutations was observed in patients with NUP98 translocations [185]. High-level transcriptional expression of *NUP98-HOX* correlated with higher transcript levels of *FLT3* and an increased incidence of FLT3 activating mutations [185]. A novel model combining an FLT3-ITD mutation with NHD13 (HOXD13) was generated using their respective endogenous promoters [186]. Initially, these transgenic mice developed leukemia with both primitive myeloid and lymphoid origin. Later, strictly myeloid leukemia with minimal differentiation were monitored [186]. Indeed, *NHD13* transgene enhanced the overexpression of the *HOX* genes, *HOXA7*, *HOXA9*, *HOXB4*, *HOXB6*, *HOXB7*, *HOXC4*, and *HOXC6* [186], shown to play an important role in HSC self-renewal and are upregulated in acute leukemia [187,188,189]. Nevertheless, mice encountered a spontaneous loss of heterozygosity with a high frequency, resulting in the loss of WT *FLT3* allele, [186], a characteristic of patients with FLT3-ITD mutations [180]. These transgenic mice provide a model to study the molecular pathways underlying MDS-related AML [186].

##### NPM1c+/FLT3

NPM1c+ and FLT3-ITD double mutations are found in about 40% of AML patients [190]. A compound transgenic mouse model with a double mutation in NPM1 and FLT3 was generated by crossing conditional *Npm1^flox−cA/+^* with constitutive *Flt3^ITD/+^* mice [191]. Inducing recombination of *Npm1^flox−cA^* in hematopoietic stem cells was accomplished by crossing the double heterozygous mice into *Mx1-Cre* transgenic mice [191]. Double mutant mice developed AML and died by the age of 31–68 days. Peripheral blood showed increased leukocyte counts, reduced numbers of circulating B and T lymphocytes along with a marked population of immature blasts, while BM cells exhibited increased self-renewal potential [191]. Solid organs were infiltrated with abnormal myeloid cells inducing splenomegaly and hepatomegaly by the time of death, highlighting the role of this double mutation in leukemogenesis [191].

##### N-RAS-G12D + CBFB-MYH11

A knock-in mice (*Nras^LSL-G12D^; Cbfb^56M^*) with an allelic expression of oncogenic N-RAS^G12D^ and CBFB-MYH11 developed leukemia in a cell-autonomous manner, with a short median latency and high leukemia-initiating cell activity [192]. Mice displayed an increased survival of pre-leukemic short-term HSCs and myeloid progenitor cells with a sustained blocked differentiation induced by the fusion protein [192]. *Nras^LSL-G12D^; Cbfb^56M^* leukemic cells were sensitive to pharmacologic inhibition of the MEK/ERK signaling pathway [192], highlighting the importance of this pathway in AML and proposing MEK inhibitors as potential therapeutic agents in inv16/ N-RAS^G12D^ AML [192].

##### NPM1c + N-RAS-G12D

One of the most common mutations with NPM1c+ is the *N-RAS* mutation occurring *in* 20% of NPM1c+ AML patients [190]. *NPM1* and *N-RAS* double mutant transgenic mice (*Npm1^cA/+^; Nras^G12D/+^*) developed high penetrance, enhanced self-renewal capacity in hematopoietic progenitors, and AML-like myeloid differentiation bias [193]. At the genomic level, frequent amplification of the mutant *N-RAS-G12D* allele was observed, along with other somatic mutations in AML driver genes [193]. Within the *HOX* genes, which were overexpressed, *HOXa* genes and downstream targets were crucial for the survival of the double-mutant mice [193].

##### WT1-R394W + FLT3-ITD

Wilms tumor 1 (WT1) is a zinc finger transcriptional regulator of target genes implicated in cell differentiation and quiescence [194]. Mutations in *WT1* occur in 10–15% of CN-AML, and it is frequently associated with mutations in several genes [194,195]. *FLT3-ITD* and *WT1* mutations, when present concomitantly, identify a group of AML patients that fail to respond to the standard induction chemotherapy, which results in poor OS [195,196]. Double mutant mice *Flt3*^+/ITD^/*Wt1*^+/R394W^ displayed manifestations of shortened survival, myeloid expansion in the BM, anemia, and erythroid dysplasia [197]. Although this model did not appear sufficient to consistently recapitulate human AML, it demonstrated that the combined mutations resulted in a more aggressive disease than either mutant genotype [197].

#### 3.2.7. Humanized Models

Humanized mouse models, injected with AML cell lines or patient-derived AML blasts, offered a faster approach and were instrumental in studying different aspects of AML. Several models were attempted to study AML in Nude mice with little success [198,199]. This section will focus on promising models for AML studies.

##### SCID Mice

The severe combined immuno-deficient (*SCID*) mice lacking B and T cell immunity [200], represent essential humanized AML mouse models [201]. Indeed, patient-derived AML cells engraftment enabled the identification of leukemia-initiating cells (LIC), expressing CD34^+^ CD38^−^ surface markers, recapitulating the human HSCs signature [202]. Engraftment of AMLs from different FAB classes into *SCID* mice reflected their intrinsic biologic behavior, suggesting a clinical correlation to the growth and dissemination of these leukemic subtypes [203]. However, lack of species cross-reactivity of cytokines and the innate host immunity against human AML cells resulted in poor engraftment of the BM [204]. In an attempt to overcome these limitations, exogenous human cytokines and growth factors were provided, which resulted in better engraftment of human cells [202,204,205,206]. One limitation of this model is the “leakiness” of the *SCID* mutation occurring in around 10% of the mice [207]. These mice present functional B and T cells, enhanced natural killer (NK) cell activity, and complement activation decreasing the engraftment efficiency [208]. An attempt to bypass this problem uses radiation and/or anti-asialo-GM1 antibody pretreatment. Unfortunately, it reduced the survival of the host, rendering this model unsuitable for human xenograft [209,210].

##### NOD/SCID Mice

To further improve tumor engraftment, a non-obese diabetic (NOD/*SCID*) model exhibiting further impairment of NK activity, reduced mature macrophage, and total lack of B and T cells was generated [211]. This model yielded higher engraftment rates with fewer human AML cells, yet with preserved morphological, phenotypical, and genotypical characteristics of the AML donors [212,213,214,215]. This model was used successfully in the screening for new therapeutics in AML [216]. In addition, human AML cells engraftment enabled the fractionation of LICs (CD34^+^ CD38^−^) into CD34^+^/CD71^−^/HLA-DR [217], CD34 Thy1 hematopoietic stem cells [218] and CD34/CD117 (or ckit) [219] subpopulations. Nevertheless, the NOD/*SCID* model presents the limitation by which higher engraftment rates required the supplementation of human cytokines or transplantation of growth-factor producing cells [220,221]. Moreover, long term engraftments (more than 8.5 months) were disabled due to the development of thymic lymphomas and restoration of NK cells activity during this period [211]. A variant with NOD/*SCID* background is the NSS model (N/S-S/GM/3) expressing Steel factor (SF), granulocyte macrophage-colony-stimulating factor (GM-CSF) and interleukin-3 (IL-3) human growth factors was generated [222]. NSS displayed enhanced engraftment of pre-leukemic myeloid cell cultures, as well as primary human AML samples, suggesting that the NSS mouse is a better host for at least a subset of AML samples [223].

##### NSG Mice

NOD/*SCID* mice were further immunosuppressed to generate the NOD/*SCID* b2-microglobulin null mice with a complete abolishment of the NK cell activity [224]. Importantly, a NOD/*SCID* IL2-Rγ^−/−^ or NSG model was generated by deletion or truncation of the gamma chain of IL-2R [225]. In addition to all the abnormalities of their predecessors, NSG mice possess a defective production of IL-2, IL-4, IL-7, IL-9, IL-15, and IL-21 as well as a severe impairment of the dendritic cell (DC) and their capacity to produce interferon γ (IFN-γ) upon stimulation [225,226]. Engraftment of newborn NSG mice with human CD34^+^ HSCs leads to the generation of a complete hematopoietic system, including red blood cells and platelets [226]. Studies revealed a significantly higher potential of AML cells engraftment in adult NSG mice in comparison to previous immunodeficient hosts [227,228]. Attempts to create different subtypes of AML were successful in NSGs [228]. NSG mice xenotransplanted with five well-characterized AML cell lines established AML models of particular relevance and significance to drug-sensitivity studies [228]. These models were exploited to study the in vivo potency of an Imidazoquinoxalines immunomodulatory drug, EAPB0503, and showed its specific activity in NPM1c+ AML subtype [229]. The usability of NSG model allowed the evaluation of the effect of a synthetic retinoid ST1926, or its encapsulated form in nanoparticles (ST1926-NP). El-Houjeiri et al. demonstrated that ST1926-NP is more potent in NSG injected with THP-1 cells [230]. MOLM-13-injected NSG mice showed strong efficacy to chemotherapy (cytarabine, 50 mg/kg) and 5+3 regimen of daunorubicin (1.5 mg/kg) [231]. These models enabled the in vivo tracking of UCB-NK cells, demonstrating their capability to migrate to BM and inhibit progression of human leukemia cells. Administering a low dose of human IL-15 enhanced survival of these mice, emphasizing the role of innate immunity in AML outcome [232]. In that sense, utilization of NSG model enabled the assessment of the combination of HSPC-NK cell adoptive transfer with the hypomethylating agents (HMAs), azacitidine (AZA), and decitabine (DAC). Cany et al. signified that the therapeutic combination exerted a significant delay in AML progression in these mice [233]. 

**Table 2:** A summary of generated AML Zebrafish models and their contribution to the understanding of the disease.

Zebrafish Model	Zebrafish Manipulation	Model Features and Major Findings	References
***spi-1: MYST3/NCOA2-EGFP***	Transgenic expression of human MYST3/NCOA2 fusion under the spi-1/*pu.1* promoter	First AML model in zebrafish 1.1% of transgenic fishes expressing the transgene developed AML after long latency	[29]
***hsp70: AML1-ETO***	Transgenic expression of human AML1-ETO fusion under *hsp70* promoter	A phenotype similar to human AML Disruption of definitive hematopoiesis: the switch of cell fates from erythroid to myeloid through gata1 downregulation and pu.1 overexpression AML1-ETOs effects on HPCs differentiation was mediated through Cycloxygenase-2 (COX-2) and β-catenin signaling pathways	[36,37]
***mRNA: NPMc+***	mRNAs injection into 1-cell–stage embryos followed by morpholinos (MOs) targeting *npm1a* and *npm1b*	Perturbation of primitive and definitive hematopoiesis Alterations in the expression of major transcription factors (pu.1+, mpx+, csf1r+, c-myb, CD41, RUNX1)	[52]
***HSE-MYCN-EGFP***	Induction of murine *N*-myc gene through heat-shock promoter	AML development with high incidence and rapid onset Enhancement of primitive hematopoiesis through alteration of transcription factors (pu.1, gata1, scl, lmo2, p27kip and p21cip1) Activation of major cancer signaling pathways	[41]
**IDH1/2 mutants**	Knockdown of zebrafish *idh1* and *idh2* (*zidh1* and *zidh2*) by morpholino knockdown and Transcription activator-like effector nuclease (TALEN-)mediated mutagenesis	*zidh1* suppression/deletion is correlated with a blockage of differentiation of the myeloid lineage *zidh1* effects definitive hematopoiesis exclusively *zidh2* affects primitive hematopoiesis exclusively	[63]
	Transgenic expression of human IDH1 mutation	Embryos recapitulated the features of human AML	
***FLT3-ITD-2A-EGFP spi-1: NPM1-Mut-PA spi-1:***	Transgenic expression of human FLT3-ITD or/and NPM1 mutations under the *spi-1* promoter	Myeloproliferative neoplasm (MPN) development as a result of a single mutation. 66.6% of double mutant transgenic fish showed increased precursor cells in the kidney marrow along with dedifferentiated myeloid blasts.	[53]
***spi-1: CREB-EGFP***	Expression of *CREB-EGFP* under *spi-1* promoter in myeloid lineage	Alteration of primitive hematopoiesis in embryos AML development in 79% of adult fishes by 9–14 months Aberrant expression of 20 genes diagnosed in pediatric AML	[57]
***Spi-1: SOX4-EGFP***	Expression of *SOX4* protein downstream the *spi-1* promoter	Increase in the number of myeloid progenitor cells and blast cells in the kidney marrow Distortion of the kidney structure	[59]

**Table 3:** A summary of generated AML mice models and their contribution to the understanding of the disease.

Mouse Model			Manipulation	Outcomes and Major Findings	References
**Chemically-Induced Model**			Transplantable AML models were generated using the L1210 and p388 cell lines, isolated from DBA/2 mice chemically exposed to the carcinogen 3-methylcholantrene.	Provide a platform for testing chemotherapeutic drugs, studying their kinetics, and evaluating their anti-leukemic effectiveness (mainly Cytarabine)	[83,84,90]
**Radiation- Induced Model**		**RF model**	Myeloid leukemia was developed following exposure to fission neutron irradiation or γ irradiation	FLT3-ITD mutations were identified in 10% of RF-AML mice which correlates with the occurrence of mutation of human AML	[98,100,101]
		**SJL/J model**	The radiation induced AML (RI-AML) in this model, is similar to the secondary human AML occurring after irradiation of Hodgkin disease patients	The efficient development of AML in this model was achieved by adding promoting factors, corticosteroids and growth factors like colony stimulating factor CSF-1, known to be high in AML patients	[103,104]
		**C3H/He and CBA models (CBA/Ca, CBA/Cne, and CBA/H)**	These models were generated by cross breeding Bragg albino with DBA mice	CBA model is considered the most favorable model in RI-AML High incidence of AML after exposure to radiation or benzene with lower latency compared to other models, Mimics human AML at the cytological, histopathological, and molecular levels.	[107,108,234]
**Virally-induced leukemia models MuLV**			Murine leukemia viruses (MuLV) induce non-B and non-T cell leukemia in mice	Same infection of MuLV induces several subtypes of AMLthat resembles FAB classification Identifies unknown oncogenes contributing to leukemogenesis.	[112,113,116,117] + Table 2
**Transposon models**			Sleeping Beauty (SB) transposon is another insertional mutagenesis system, allowing overexpression or inactivation of specific genes depending on the transposon orientation and integration site	Identification of mutations in leukemia genes, which provided new pathogenetic insights and potential therapeutic targets in NPM1c+ AML	[118,119,121]
**Trans-genic models**	Single mutation	**Promyelocytic Leukemia protein (PML)-RARα t(15;17)**	Expressing PML-RARα under *CD11b* promoter	Abnormal myelopoiesis and increased radiation sensitivity No AML development	[124]
			Expressing PML-RARα under human cathepsin G (*HCG*) promoter	APL phenotype after long latency period Remission seen after All Trans Retinoic Acid (ATRA) treatment in APL	[125]
			Expressing PML-RARα under human MRP8 (*hMRP8*) promoter	APL phenotype after long latency period Remission seen after ATRA treatment in APL	[126]
		**AML1- Eight-Twenty One oncoprotein (ETO)**	Knock-in of AML1-ETO into mouse embryos (AML1-ETO/+)	Absence of liver-derived definitive hematopoiesis Embryonic lethality	[127,128]
			Expressing AML1-ETO in adult bone marrow progenitor cells	Abnormal maturation and proliferation of progenitor cells No AML development	[130,131]
			Expressing AML1-ETO under human MRP8 (hMRP8) promoter	AML development after exposure to *N*-ethyl-*N*-nitrosourea	[132]
		**CBFB-MYH11**	Knock-in embryonic mice (Cbfb+/Cbfb-MYH11)	Lack of definitive hematopoiesis Embryonic lethality	[138]
			Chemical/ retroviral mutagens on heterozygous CBFB-MYH11 adults	AML development	[138,139]
			Conditional knock-in adult mice (*Cbfb+/56M)*	AML development in 90% of mice after 5 months	[140]
		**Mutant Nucleophosmin-1 (NPM1c+)**	Knock-in mice expressing NPM1 with mutation A (NPM1c+)	Homozygotes encountered embryonic lethality 1/3 of the heterozygotes (*Npm1wt/c+*) developed fetal myeloproliferative disease but not AML	[143]
			Expression of NPM1 with mutation A (NPM1c+) under the pCAG promoter	1/3 of the transgenic mice developed leukemia after a long period of latency	[144]
			Expression of humanized NPM1c+ in the hematopoietic stem cells	*HOX* overexpression Enhanced self-renewal Expanded myelopoiesis	[121]
		**Fms-related tyrosine kinase 3 internal tandem repeats (FLT3-ITD)**	Expressing FLT3-ITD under the vav hematopoietic promoter	Myeloproliferative syndrome (MPS) Megakaryocytic hyperplasia and thrombocytosis No AML development	[146]
			FLT3-ITD knock-in mice with lost FLT3 wild-type allele	Myeloid expansion and aggressiveness of the MPS disease No AML development	[147]
		**Mixed Lineage Leukemia (MLL)**	Embryonic stem cell formed by in-frame fusion of AF9 with exon 8 of mouse MLL	AML development	[152]
			Conditional expression of MLL-AF9 using programmed interchromosomal recombination	AML development	[153]
			Conditional expression of MLL-AF9 in LT-HSC	Aggressive AML Extensive tissue infiltration Chemoresistance Expression of genes related to epithelial-mesenchymal transition (EMT) in solid cancers	[154]
			Early introduction of MLL	Abnormalities of myeloid cell proliferation and differentiation	[155]
		**IDH 1/2**	Expressing IDH1/2 under the vav promoter (Vav-KI mice) or specifically in cells of the myeloid lineage (LysM-KI mice)	Increased number of early hematopoietic progenitors Splenomegaly Anemia Extramedullary hematopoiesis, characteristics of a dysfunctional BM niche and partial blockage in myeloid differentiation Induction of leukemic DNA methylation signature in mouse model	[167]
	Compound mutations	**K-RAS-G12D + PML-RARα**	Constitutive expression of K-RAS and PML-RARα	Rapid-onset and highly penetrant, lethal APL-like disease	[170]
		**N-RAS12D + BCL-2**	*MMTVtTA /TBCL-2/NRASD12* Expression of hBCL2 in a primitive compartment by mouse mammary tumor virus–long terminal repeat	MDS development Expanded leukemic stem cell (Lin^−^/Sca-1^+^/c-Kit^+^) populations Increased apoptosis Malignant disease with a penetrance of around 80% and a latency period of 3 to 6 months	[175]
			*MRP8 [BCL-2/NRASD12]* Constitutive expression *of BCL-2* under human *MRP8* promoter	AML development Expanded leukemic stem cell (Lin^−^/Sca-1^+^/c-Kit^+^) populations No apoptotic cells Malignant disease with a penetrance of around 80% and a latency period of 3 to 6 months	[175]
		**MLL-PTD + FLT3-ITD**	Expressing MLL-PTD and FLT3-ITD under their respective endogenous promoters	Latent AML with a short life span, extensive extramedullary involvement and increased aggressiveness Normal chromosomal structures Reduced *MLL*-WT expression Loss of *FLT3*-WT and increased total *FLT3* expression Increased *HOXA9* transcript levels	[179]
		**NUP98-HOXD13 + FLT3-ITD**	Expressing FLT3-ITD and NHD13 (HOXD13) under their respective endogenous promoters	Myeloid leukemia with minimal differentiation Overexpression of several *HOX* genes Spontaneous loss of heterozygosity with a high frequency, resulting in the loss of WT *FLT3* allele	[186]
		**NPM1c+ - FLT3**	Crossing conditional *Npm1^flox−cA/+^* with constitutive *Flt3^ITD/+^* mice	AML development Lethality by the age of 31-68 days Modified blood cell counts Immature blasts in BM Myeloid cells infiltration into organs Splenomegaly and hepatomegaly	[191]
		**N-RAS-G12D + CBFB-MYH11**	Allelic expression of oncogenic N-RAS^G12D^ and CBFB-MYH11	Leukemia development in a cell-autonomous manner with a short median latency High leukemia-initiating cell activity Increased survival of pre-leukemic short-term HSCs and myeloid progenitor cells with blocked differentiation Leukemic cells were sensitive to MEK/ERK inhibitors	[192]
		**NPM1c + N-RAS-G12D**	Conditional expression of *NPM1c+* and *N-RAS-G12D*	AML-like myeloid differentiation bias Hematopoietic progenitors with high penetrance and enhanced self-renewal capacity Frequent amplification of the mutant *N-RAS-G12D* allele Somatic mutations in AML driver genes Overexpression of *HOX* genes	[193]
		**WT1-R394W + FLT3-ITD**	Crossing *Flt3^+/ITD^* mice with *Wt1^+/R394W^* mice	MDS/MPN development Shortened survival Myeloid expansion in the BM, Anemia Erythroid dysplasia	[197]
**Xenograft/humanized models**		***SCID* mice**	Autosomal recessive mutation	Lack of B and T cells Retained innate immunity and cytokines Identification of leukemia initiating cells (LIC) Poor engraftment of human AML cells in the BM	[200]
		**NOD/*SCID* mice**	NOD/*SCID* model: Express additional mutations	Impairment of NK activity Reduced mature macrophages Total lack of B and T cells Fractionation of LIC into subpopulations	[211]
			NSS model (N/S-S/GM/3): variant of NOD/*SCID* mice expressing SF, GM-CSF and IL-3	Better host for a subset of AML	[222,223]
		**NSG mice**	Deletion or truncation of the γ chain of IL-2R	Defective production of major interleukins and IFN-γ Impairment of dendritic cells Complete abolishment of the NK cell activity Higher engraftment capacity of human AML cells than previous models	[224]

**Table 4:** **Murine leukemia virus** (MuLV) induced AML models: Major gene discoveries and their involvement in different French–American–British (FAB) AML subtypes.

MuLV Virus	Mouse Strain	AML Subtype	FAB Classification	Major Gene Discoveries	References
CasBrM-MuLV	NFS	Granulocytic	M1 or M2	*His-1*	[235,236]
CasBrE MuLV	NIH Swiss	Myeloid	M1 or M2	*Fli-1*	[237,238,239]
Endogenous ecotropic MuLV	AKXD-23	Granulocytic	M1 or M2	*Evi-1*	[240,241]
Friend-MuLV	C57BL/6	Granulocytic	M1 or M2	*Ccnd1*	[237,242,243]
Friend-MuLV	DBA/2	Myeloblastic	M1 or M2	*Evi-1, & c-myb*	[244,245,246]
M-MuLV	BALB/c	Promonocytic	M5	*c-myb*	[246,247]
B ecotropic MuLV	BXH-2	Myelomonocytic	M4	*c-myb*, *HOXa7*, *HOXa9*, *Meis1*, *CBFa1*, *SOX4*, *Hhex*, *Rarg*, *Sharp1*, *Ccnd3*, *Cdc25l*, *RASGRP*, *Clabp*, *Hmgcr*, *Nf1*, & *Il17r*	[248,249,250,251,252,253,254,255]

## 4. Drosophila Melanogaster

### AML1-ETO

The chromosomal translocation t(8:21)(q22;q22) is frequent and common in AML. It represents up to 40% of AML subtype M2 of the FAB classification [256]. The fusion gene resulting in this translocation encodes for the chimeric protein AML1-ETO, which contains the N-terminus of AML1 (including its DNA binding domain) and most of the ETO protein [33,257], and inhibits the expression of AML1 target genes leading to leukemogenesis [258]. The detailed molecular mechanism governing this interference is poorly understood, which enticed the generation of several animal models to understand its mode of action. AML1-ETO alone is not sufficient to induce leukemia unless accompanied by secondary mutations [130,131,259]. The simplicity of genetics and ease of manipulation in *Drosophila* presents it as an attractive model to study this complex translocation. In addition, *Drosophila* hematopoiesis is comparable to that of mammals [260]. Two AML1-ETO models of genetically engineered *Drosophila* were generated. In the first model, AML1-ETO is a constitutive transcriptional repressor of AML1 target genes. In the second model, AML1-ETO dominantly interferes with AML1 activity by potentially competing for a common co-factor [261]. The transcription factor Lozenge (Lz) that is similar to human AML1 protein is necessary for the development of crystal cells, one of the major *Drosophila* blood cells, during hematopoiesis [262]. Using these models and by comparison with loss-of-function phenotypes of Lz, AML-1-ETO was shown to act as a constitutive transcriptional repressor [261]. Osman et al. reported that AML1-ETO inhibits the differentiation of crystal cell lineage, and induces an increase in the number of circulating LZ+ progenitors. Moreover, large scale RNA interference screen for suppressors of AML1-ETO in vivo showed that *calpainB* is required for AML1-ETO-induced leukemia in *Drosophila*. Surprisingly, calpainB inhibition in Kasumi-1 cells (AML patient cell line carrying t(8;21) translocation) leads to AML1-ETO degradation and impairs their clonogenic potential [263]. Another study identified pontin/RUVBL1as a suppressor of AML1-ETO. Indeed, PONTIN knock-down inhibits the proliferation of t(8;21) positive cells, and that PONTIN is essential for Kasumi-1 clonogenic potential and cell cycle progression [264]. Thus, AML1-ETO can be recapitulated in Drosophila blood for investigating its mechanism and identifying potential targeted therapeutics for this AML subtype.

Despite advances in our understanding of many molecular mechanisms, in vitro research falls short in determining overall effect of treatment modalities or drug discovery. AML is an intricate disease where culture consisting of a single cell line system, can never recapitulate the complexity of the disease. In the difficulty of obtaining primate models of AML, small rodents, zebrafish, and Drosophila with well characterized genetic background and relative ease of manipulation, are the backbone of current work where leukemic cells are interfaced with the host immunity, metabolic environment and importance of the niche ation. Not one model is sufficient to address all posed questions. However, collectively, these models have expanded our knowledge and understanding of several pathways and important players in AML pathogenesis.

## Figures and Tables

**Table 1:** 2017 European LeukemiaNet (ELN) prognostic groups according to genetic abnormalities of acute myeloid leukemia (AML) [12].

Prognostic Group	Genetic Mutations and Abnormalities
Favorable	t(8;21)/RUNX1-RUNX1T1 inv(16) or t(16;16)/*CBFB-MYH11* Mutated *NPM1* without *FLT3*-ITD or with *FLT3*-ITD ^low^ * Biallelic mutated *CEBPA*
Intermediate	Mutated *NPM1* and *FLT3*-ITD ^high^ * Wild-type *NPM1* without *FLT3*-ITD or with *FLT3*-ITD ^low^ * t(9;11)/MLLT3-KMT2A Cytogenetic abnormalities not classified as favorable or adverse
Adverse	t(6;9)/ DEK-NUP214 t(v;11q23.3)/*KMT2A* rearranged t(9;22)/*BCR*-*ABL1* inv(3) or t(3;3)/*GATA2*,*MECOM(EVI1)* Complex karyotype Monosomal karyotype Wild-type *NPM1* and *FLT3*-ITD ^high^ * Mutated *RUNX1* Ϯ Mutated *ASXL1* Ϯ Mutated *TP53*

* ^Low^, low allelic ratio (<0.5); ^high^, high allelic ratio (>0.5); Ϯ these mutations should not be used as an adverse prognostic marker if they co-occur with favorable-risk AML subtypes.
